# Anesthesia With Conscious Sedation for Balloon Dilatation in the Setting of Severe Subglottic Tracheal Stenosis: A Case Report

**DOI:** 10.7759/cureus.79488

**Published:** 2025-02-22

**Authors:** Jose E Solache-May, Victor M Ayuso-Diaz, Valentina Magos-Gamboa, Maria E Ayuso-Diaz, Angelica Moreno-Enriquez

**Affiliations:** 1 Anesthesiology, Hospital Regional de Alta Especialidad de Yucatán (HRAEPY) Universidad Autónoma de Yucatán, Yucatan, MEX; 2 Clinical Recruitment, Medical Care and Research, Yucatán, MEX; 3 Surgery, Elvia Carrillo Puerto Regional Hospital, Yucatan, MEX; 4 Genomic-Metabolic Unit, Marist University of Mérida, Yucatan, MEX; 5 Anesthesiology, Hospital Regional Elvia Carrillo Puerto, Yucatan, MEX

**Keywords:** advanced sedoanalgesia, airway management, awake anesthesia, subglottic tracheal stenosis, tracheal balloon dilatation

## Abstract

Severe subglottic tracheal stenosis represents a significant clinical challenge due to its impact on respiratory function and the inherent complexity of airway management. In procedures such as tracheal balloon dilatation, the anesthetic approach plays a crucial role in ensuring patient safety and comfort while minimizing perioperative risks. This case describes the anesthetic management of a conscious patient undergoing this procedure, highlighting the strategies employed and the advantages of this technique in difficult airway situations.

## Introduction

Anesthetic planning in patients with difficult airways is a major challenge in clinical practice, particularly in the context of conditions such as severe subglottic tracheal stenosis [1,2]. This condition, characterized by a critical narrowing of the tracheal lumen, can complicate ventilation and increase the risk of airway management during surgery [3]. It requires highly specialized strategies to optimize ventilation and minimize risk during complex surgical procedures, including the use of advanced airway techniques and specific anesthetic agents [4]. The use of anesthesia in conscious patients, often referred to as “conscious sedation,” is a safe and effective option in this scenario, preserving spontaneous breathing and reducing complications associated with invasive airway instrumentation [5,6]. In procedures, such as tracheal balloon dilatation, this technique offers significant advantages, including improved hemodynamic control, reduced exposure to deep anesthetic agents, avoidance of positive pressure ventilation, and lower rates of postoperative respiratory complications [7,8].

## Case presentation

**Figure 1:**
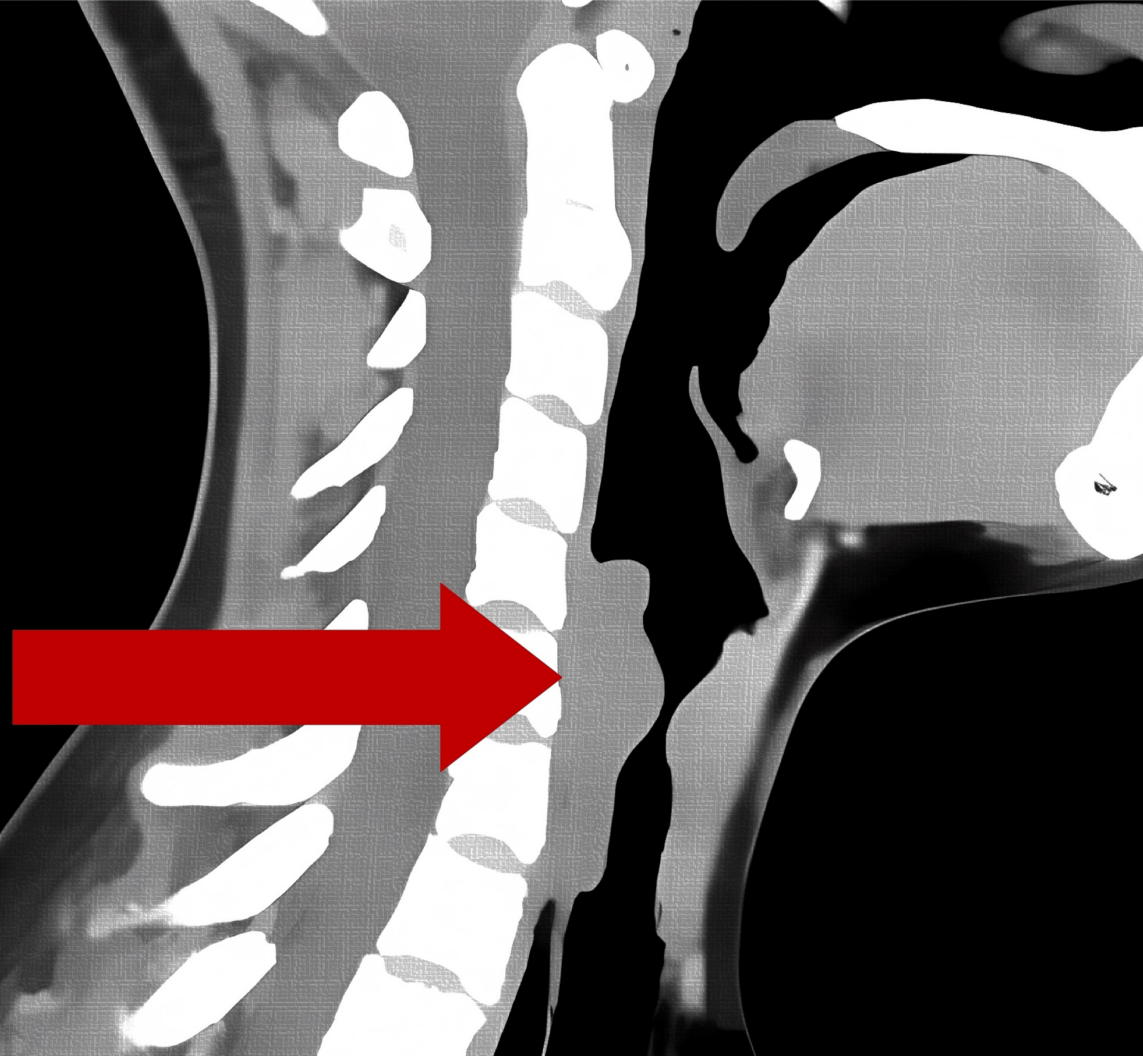
Computed tomography (CT) scan of the neck and chest showing severe tracheal stenosis with a luminal diameter of 5 mm. The imaging studies allowed the severity of the obstruction to be assessed and the appropriate surgical intervention to be planned.

A 24-year-old male patient from Valladolid, Yucatán, diagnosed with severe subglottic tracheal stenosis, was scheduled for elective balloon tracheal dilation. The patient, with a history of childhood asthma controlled with salbutamol and no current medications, sustained a traumatic brain injury in a road traffic accident in April 2024. This injury required invasive mechanical ventilation and a seven-day stay in intensive care. After extubation, the patient developed dyspnea and stridor within 48 hours, which initially improved with supplemental oxygen via nasal prongs at 3 L/min. A computed tomography scan of the neck and chest revealed severe tracheal stenosis with a luminal diameter of 5 mm (Figure 1), secondary to the trauma sustained during the accident rather than complications of positive pressure ventilation.

Pre-operative assessment in the anesthesia unit showed that the patient was in good general condition, conscious, and oriented. His vital signs were stable: body mass index (BMI) of 30.0 kg/m², blood pressure of 110/70 mmHg, heart rate of 72 bpm, respiratory rate of 16 breaths/min, temperature of 36.3°C, and oxygen saturation of 99%. Airway assessment revealed a Mallampati score of II and a sternomental distance of 12.5 cm. Preoperative laboratory tests (Table 1) were within normal limits, confirming no contraindications to the planned procedure.

**Table 1:** Laboratory results prior to surgery AST: aspartate aminotransferase, ALT: alanine transaminase

Examination	Result	Reference range
Blood group and type	O+	O+, A+, B+, AB+
Hemoglobin (Hb)	12.8 g/dL	13.0–17.0 g/dL (male)
Hematocrit (Ht)	39.40%	40%–54% (male)
Leukocytes (WBC)	9.4 x 10⁹/L	4.0–11.0 x 10⁹/L
Platelets	139,000/µL	150,000–450,000/µL
Prothrombin time (PT)	12.1 s	11.0–14.0 s
International normalized ratio (INR)	1.06	0.9–1.2
Partial thromboplastin time (PTT)	34.0 s	25–35 s
Creatinine (Cr)	0.7 mg/dL	0.6–1.2 mg/dL
Urea	30 mg/dL	10–50 mg/dL
Glucose (Gluc)	77.6 mg/dL	70–99 mg/dL
Sodium (Na)	140 mmol/L	135–145 mmol/L
Potassium (K)	3.12 mmol/L	3.5–5.0 mmol/L
Chloride (Cl)	103 mmol/L	98–106 mmol/L
Total bilirubin (BT)	0.6 mg/dL	0.1–1.2 mg/dL
Direct bilirubin (BD)	0.2 mg/dL	0.0–0.3 mg/dL
Indirect bilirubin (BI)	0.4 mg/dL	0.2–0.7 mg/dL
Total proteins	6.1 g/dL	6.0–8.0 g/dL
Albumin	3.9 g/dL	3.5–5.0 g/dL
AST (SGOT)	27.1 U/L	10–40 U/L
ALT (SGPT)	39.2 U/L	7–56 U/L

The surgery was performed under general anesthesia with the patient awake according to a sedoanalgesia protocol. The anesthetic plan included premedication with intravenous dexmedetomidine (0.7 mcg/kg/h) administered 20 minutes before admission to the operating theatre. After checking the functionality of the anesthesia and suction equipment, standard monitoring was started (non-invasive blood pressure (NIBP), pulse oximeter, ECG, end-tidal CO₂ (EtCO₂)). The patient's initial vital signs included a heart rate of 56 BPM, respiratory rate of 14 RPM, blood pressure of 121/77 mmHg, and oxygen saturation of 100% with supplemental oxygen at 5 L/min via a fenestrated mask (Table 2).

**Table 2:** Patients' initial vital signs compared to reference values Patients' initial vital signs compared to reference values oxygenation parameters (SpO₂ and FiO₂) were optimal when supplemental oxygen was administered.

Parameter	Case value	Reference range
Heart rate (HR)	56 BPM	60-100 BPM
Respiratory rate (RR)	14 RPM	12-20 RPM
Blood pressure (BP)	121/77 mmHg	90/60 - 120/80 mmHg
Oxygen saturation (SpO₂)	100%	≥ 95%
End-tidal CO₂ (EtCO₂)	17 mmHg	35-45 mmHg
Fraction of inspired oxygen (FiO₂)	97%	21%-100% (depending on oxygen therapy)

For anesthetic management, dexmedetomidine infusion was started at 0.4 mcg/kg/h. Three minutes into the procedure, remifentanil was started using iTIVA simulation software (iTIVA, Cali, Colombia) guided by the Minto model, targeting a site of action concentration of approximately 5 ng/mL. 0.2 mg of glycopyrrolate was administered intravenously to improve surgical visualization and minimize secretions. Recognizing that sedoanalgesia alone would not be sufficient to suppress airway reflexes during instrumentation, bilateral superior laryngeal nerve blocks and a transtracheal block with 2% lidocaine were performed to ensure effective airway anesthesia and prevent coughing or laryngospasm. The surgical team used a flexible bronchoscope and a video laryngoscope to carefully navigate the stenosed airway during balloon dilatation.

Continuous monitoring of hemodynamic and respiratory parameters was maintained throughout the procedure. The patient's hemodynamic status remained stable, and a transient episode of sinus bradycardia was effectively managed by reducing the dexmedetomidine infusion to 0.3 mcg/kg/h. The patient remained responsive and cooperative, with a Richmond Agitation Sedation Scale (RASS) score of -2 and a Ramsay score of 3. Vital signs at this time (Table 3) reflected adequate cardiovascular and respiratory function. This integrated approach - combining targeted regional airway blocks with precisely titrated sedoanalgesia - ensured safe airway instrumentation and optimal procedural conditions, ultimately contributing to a successful outcome.

**Table 3:** Vital signs during the procedure During the procedure, the patient remained hemodynamically stable except for a brief episode of sinus bradycardia, which was managed by adjusting the dexmedetomidine infusion. Oxygenation levels were optimal with supplemental oxygen. However, the EtCO₂ value of 0 mmHg suggests either transient apnea, airway occlusion or capnography malfunction and requires further clinical correlation.

Parameter	Case value	Reference range
Heart rate (HR)	72 BPM	60-100 BPM
Respiratory rate (RR)	12 RPM	12-20 RPM
Blood pressure (BP)	130/74 mmHg	90/60-120/80 mmHg
Oxygen saturation (SpO₂)	100%	≥ 95%
End-tidal CO₂ (EtCO₂)	0 mmHg	35-45 mmHg
Fraction of inspired oxygen (FiO₂)	100%	21%-100% (depending on oxygen therapy)

In addition, 30 mg IV ketorolac and 8 mg IV dexamethasone were administered for pain and inflammation management. Drug infusions were stopped at the end of surgery. The total operative time was 58 minutes, and the anesthetic time was 68 minutes. Three hundred ml of Hartmann's solution was administered, with minimal bleeding and unquantified uremia. At the end of the surgery, the patient recovered adequately from anesthesia with no immediate complications. Continuous monitoring of vital signs was stable, with oxygen saturation within normal parameters. Supplemental oxygen continued to be administered via a fenestrated mask at 5 L/min to ensure adequate oxygenation during the immediate recovery period. Due to the nature of the procedure and the patient's previous condition, particular attention was paid to monitoring the respiratory pattern, with constant monitoring for signs of respiratory failure. As a result, the patient met the anesthetic discharge criteria and was favorably discharged from the post-anesthetic recovery area. Appropriate instructions were given for his post-operative management, and follow-up visits were scheduled to ensure correct clinical evolution and ongoing assessment of long-term respiratory function.

This case highlights the importance of anesthetic management in patients with severe subglottic tracheal stenosis, where the decision to keep the patient awake during the procedure was based on the need to maintain airway patency and reduce the risk of complications associated with tracheal intubation. The technique of regional anesthesia with sedoanalgesia allowed adequate control of the airway, ensured patient cooperation, and minimized the risk of cannula dislodgement or injury to the stenosed area. The decision to use sedoanalgesia was also motivated by the clinical condition of the patient, who had experienced a significant post-traumatic complication following a road traffic accident requiring advanced airway management. Preservation of respiratory function and hemodynamic stability were priorities in the anesthetic planning. In addition, this technique allowed constant monitoring of the patient with rapid intervention in the event of any change in respiratory or hemodynamic parameters. This approach demonstrates how an appropriate choice of anesthetic technique, together with personalized management, can optimize safety and recovery in complex procedures such as tracheal dilation in patients with severe subglottic stenosis.

## Discussion

Severe subglottic tracheal stenosis is a complex clinical condition that poses significant challenges for diagnosis, and surgical and anesthetic management. This pathology, characterized by narrowing of the lower airway, can result from a variety of factors including direct trauma, prolonged intubation, postoperative complications, or chronic inflammatory disease. In particular, high pressures in the cuff of the endotracheal tube during ICU ventilation have been strongly associated with the development of tracheal stenosis, making it a critical quality issue. Maintaining appropriate cuff pressures and considering early tracheostomy are essential techniques to reduce the incidence of this serious complication. The diagnosis of subglottic tracheal stenosis is made by a detailed clinical evaluation, including a thorough medical history and advanced imaging studies such as computed tomography or bronchoscopy, which allow direct visualization of the affected tracheal anatomy [1,2]. In terms of pathophysiology, chronic inflammation, and resultant structural damage leads to fibrosis and collapse of the tracheal walls, making both ventilation and airway instrumentation difficult during surgical procedures. According to the literature, the anesthetic management of these patients is complex and requires a comprehensive understanding of the underlying pathology together with the implementation of strategies to minimize the risk of respiratory complications during surgery [2-4].

The decision to keep the patient awake during tracheal balloon dilatation under sedoanalgesia is mainly justified by the preservation of respiratory function and responsiveness to any airway compromise. This strategy is particularly important in patients with severe tracheal stenosis, as traditional general anesthesia with endotracheal intubation may significantly increase the risk of airway obstruction or postoperative respiratory dysfunction [5,6]. Several studies have shown that awake anesthesia provides a superior level of airway control by allowing the patient to maintain spontaneous breathing, which facilitates immediate postoperative recovery and reduces the possibility of serious respiratory complications [7].

The literature presents sedoanalgesia as an effective therapeutic option for the management of these procedures. The use of sedatives such as dexmedetomidine in combination with fast-acting opioids such as remifentanil allows a balance between sedation and pain control without compromising the patient's respiratory function [8,9]. In particular, dexmedetomidine has been shown to provide sedation and analgesia without significant respiratory depression, resulting in a lower incidence of respiratory complications in patients with tracheal stenosis [8].

Continuous perfusion of remifentanil, guided by local concentration, is another strategy that allows precise control of intraoperative analgesia without causing excessive sedation or respiratory depression. The use of pharmacokinetic models such as the iTIVA system for remifentanil dosing has proven effective in adjusting the depth of anesthesia in real-time, which is critical in procedures where respiratory stability is essential [10]. However, delivering oxygen to a patient with severe tracheal stenosis using positive pressure ventilation poses significant challenges; even small amounts of blood or saliva can cause complete airway obstruction. It is therefore imperative that equipment for emergency cricothyroidotomy, micro-laryngeal tubes, and jet ventilation is readily available to manage any apnea resulting from inadvertent oversedation. Choosing sedoanalgesia while the patient is awake also facilitates cooperation by allowing the maintenance of spontaneous breathing - a key factor in avoiding airway collapse or complete obstruction [1,4,6]. In the event of airway complications, the patient can be rapidly assessed and managed with minimal intervention, significantly reducing the risks associated with intubation and mechanical ventilation [3].

Despite the benefits of sedoanalgesia, it is important to recognize and be prepared for potential complications that may occur during airway sharing during these procedures. In addition to the risks of positive pressure ventilation in patients with severe tracheal stenosis, complications such as airway obstruction by blood, secretions, or reflex-induced laryngospasm may occur. Close continuous monitoring is essential to reduce these risks. Vital signs - heart rate, blood pressure, and oxygen saturation - must be monitored in real-time, and the respiratory pattern should be carefully assessed. In this context, continuous EtCO₂ monitoring and advanced blood gas analysis are invaluable tools to detect early signs of respiratory compromise and promptly adjust anesthetic interventions [11]. In addition, the team must be prepared for emergency airway management; equipment for emergency cricothyroidotomy, micro-laryngeal tubes, and jet ventilation should be readily available to manage any unexpected apnoea or airway obstruction. Compared with traditional general anesthesia, awake sedoanalgesia significantly reduces the risk of serious complications, such as acute respiratory distress syndrome or complete tracheal obstruction, and facilitates faster, less invasive recovery with earlier extubation or transfer to the post-anesthesia care unit [12]. This comprehensive and proactive approach, emphasizing continuous monitoring and readiness to intervene, is essential to ensure patient safety throughout the procedure.

## Conclusions

The anesthetic management of a patient with severe subglottic tracheal stenosis represents a significant clinical challenge, given the complexity of maintaining respiratory stability during airway-compromising procedures. This case underscores the importance of a personalized approach based on awake sedoanalgesia, which preserves spontaneous breathing and minimizes the risks associated with intubation and general anesthesia. However, the risk of apnea from oversedation remains a critical concern; therefore, continuous monitoring of respiratory parameters and careful titration of sedative agents is imperative to prevent excessive respiratory depression. The deliberate selection of agents, such as dexmedetomidine and remifentanil - administered with the aid of advanced pharmacokinetic modeling - illustrates the value of innovative strategies in managing complex patients. The documentation of this case aims to expand the current body of evidence and provide valuable insights into the safe and effective anesthetic management of airway pathology, emphasizing the need for a multidisciplinary and vigilant approach in high-risk scenarios.
